# Supplementary material for: Precise mapping of single-stranded DNA breaks by sequence-templated erroneous DNA polymerase end-labelling
Source: Nat Commun. 2025 Aug 4;16:7130. doi: 10.1038/s41467-025-62512-4 (PMC12322144; doi:10.1038/s41467-025-62512-4)
Supplement: Supplementary file 1 — Supplementary Information [file 41467_2025_62512_MOESM1_ESM.pdf]

```
atgcatcaccatcaccatcacgaaaacctgtatttcagggcatggttcagatccccaaaatccacttatccttgtagat
M  H  H  H  H  H  H  E  N  L  Y  F  Q  G  M  V  Q  I  P  Q  N  P  L  I  L  V  D
ggttcatcttatctttatcgcgcatatcacgcgtttcccccgctgactaacagcgcaggcgagccgaccggtgcgatgtat
G  S  S  Y  L  Y  R  A  Y  H  A  F  P  P  L  T  N  S  A  G  E  P  T  G  A  M  Y
ggtgtcctcaacatgctgcgcagtctgatcatgcaatataaaccgacgcatgcagcggtggtctttgacgccaagggaaaa
G  V  L  N  M  L  R  S  L  I  M  Q  Y  K  P  T  H  A  A  V  V  F  D  A  K  G  K
acctttcgtgatgaactgtttgaacattacaaatcacatcgcccgccaatgccggacgatctgcgtgcacaaatcgaaccc
T  F  R  D  E  L  F  E  H  Y  K  S  H  R  P  P  M  P  D  D  L  R  A  Q  I  E  P
ttgcacgcgatggttaaagcgatgggactgccgctgctggcggtttctggcgtagaagcggacgacgttatcggtactctg
L  H  A  M  V  K  A  M  G  L  P  L  L  A  V  S  G  V  E  A  D  D  V  I  G  T  L
gcgcgcgaagccgaaaaagccgggcgtccggtgctgatcagcactggcgataaagatatggcgcagctggtgacgccaaat
A  R  E  A  E  K  A  G  R  P  V  L  I  S  T  G  D  K  D  M  A  Q  L  V  T  P  N
attacgcttatcaataccatgacgaataccatcctcggaccggaagaggtggtgaataagtacggcgtgccgccagaactg
I  T  L  I  N  T  M  T  N  T  I  L  G  P  E  E  V  V  N  K  Y  G  V  P  P  E  L
atcatcgatttcctggcgctgatgggtgactcctctgataacattcctggcgtaccgggcgtcggtgaaaaaaccgcgcag
I  I  D  F  L  A  L  M  G  D  S  S  D  N  I  P  G  V  P  G  V  G  E  K  T  A  Q
gcattgctgcaaggtcttggcggactggatacgctgtatgccgagccagaaaaaattgctgggttgagcttccgtggcgcg
A  L  L  Q  G  L  G  G  L  D  T  L  Y  A  E  P  E  K  I  A  G  L  S  F  R  G  A
aaaacaatggcagcgaagctcgagcaaaacaaagaagttgcttatctctcataccagctggcgacgattaaaaccgacgtt
K  T  M  A  A  K  L  E  Q  N  K  E  V  A  Y  L  S  Y  Q  L  A  T  I  K  T  D  V
gaactggagctgacctgtgaacaactggaagtgcagcaaccggcagcggaagagttgttggggctgttcaaaaagtatgag
E  L  E  L  T  C  E  Q  L  E  V  Q  Q  P  A  A  E  E  L  L  G  L  F  K  K  Y  E
ttcaaacgctggactgctgatgtcgaagcgggcaaatggttacaggccaaagggcaaaaccagccgcgaagccacaggaa
F  K  R  W  T  A  D  V  E  A  G  K  W  L  Q  A  K  G  A  K  P  A  A  K  P  Q  E
accagtgttgcagacgaagcaccagaagtgacggcaacgacaggcagcaccgggtcgactgggagtacgggttccatgtct
T  S  V  A  D  E  A  P  E  V  T  A  T  T  G  S  T  G  S  T  G  S  T  G  S  M  S
aagtttacatggaaagagttaattcaattaggcagtccatcgaaagcatacgagtcctcattagcttgtatcgcacatatt
K  F  T  W  K  E  L  I  Q  L  G  S  P  S  K  A  Y  E  S  S  L  A  C  I  A  H  I
gatatgaatgcgttcttcgcccaggtggagcagatgcgttgtggcctgtctaaggaggatcccgtagtatgcgttcagtgg
D  M  N  A  F  F  A  Q  V  E  Q  M  R  C  G  L  S  K  E  D  P  V  V  C  V  Q  W
aacagcatcattgcggtgtcttatgctgctcgcaaatacggcatctcccgtatggacaccatccaggaggctctgaagaaa
N  S  I  I  A  V  S  Y  A  A  R  K  Y  G  I  S  R  M  D  T  I  Q  E  A  L  K  K
tgctcgaacttaatccctattcatacggccgtcttcaagaaaggagaagatttctggcagtaccatgatgggtgtgggtcg
C  S  N  L  I  P  I  H  T  A  V  F  K  K  G  E  D  F  W  Q  Y  H  D  G  C  G  S
tgggtacaggacccgcgaagcaaatctcggtcgaggatcacaaggtttcactggagccctatcgtcgtgaatcacgcaag
W  V  Q  D  P  A  K  Q  I  S  V  E  D  H  K  V  S  L  E  P  Y  R  R  E  S  R  K
gcgcttaaaatcttcaagtcggcatgcgatttggtagagcgtgcctctattgacgaggtattccttgacttgggacgtatc
A  L  K  I  F  K  S  A  C  D  L  V  E  R  A  S  I  D  E  V  F  L  D  L  G  R  I
tgctttaacatgttaatgtttgacaatgagtacgaattgacaggggacttaaagttaaaagatgcactgtctaatattcgc
C  F  N  M  L  M  F  D  N  E  Y  E  L  T  G  D  L  K  L  K  D  A  L  S  N  I  R
gaagcctttatcgggggaattatgatattaactcgcatttaccgcttattcctgagaaaattaagagcttgaagtttgag
E  A  F  I  G  G  N  Y  D  I  N  S  H  L  P  L  I  P  E  K  I  K  S  L  K  F  E
ggggatgtttttaatcccgaaggccgtgacctgatcaccgactgggacgacgtgattcttgcacttgggagccaggtttgc
G  D  V  F  N  P  E  G  R  D  L  I  T  D  W  D  D  V  I  L  A  L  G  S  Q  V  C
aaaggtattcgcgacagtattaaagacatcttgggctatacaacctcatgcgggctttcatcaacgaaaaacgtctgtaaa
K  G  I  R  D  S  I  K  D  I  L  G  Y  T  T  S  C  G  L  S  S  T  K  N  V  C  K
cttgcttcaaactataagaagcctgacgcccagactattgtcaagaatgactgtcttctggattttttggactgcggaaag
L  A  S  N  Y  K  K  P  D  A  Q  T  I  V  K  N  D  C  L  L  D  F  L  D  C  G  K
ttcgagattacatcctttggacgctgggtggagtcttgggaaaggaactgattgatgtccttgacttacctcatgagaac
F  E  I  T  S  F  W  T  L  G  G  V  L  G  K  E  L  I  D  V  L  D  L  P  H  E  N
tcgatcaaacacattcgtgagacatggcctgacaacgccggacagttgaaggagtttctggacgccaaggtcaaacaatct
S  I  K  H  I  R  E  T  W  P  D  N  A  G  Q  L  K  E  F  L  D  A  K  V  K  Q  S
gattatgatcgctcgacctctaacatcgacccttgaaaaccgctgatctggccgaaaagctttttaaactttcgcgcggt
D  Y  D  R  S  T  S  N  I  D  P  L  K  T  A  D  L  A  E  K  L  F  K  L  S  R  G
cgttacggacttccattatcttcacgtccggttgttaagtctatgatgtccaacaaaaacctgcgtggtaagtcgtgcaat
R  Y  G  L  P  L  S  S  R  P  V  V  K  S  M  M  S  N  K  N  L  R  G  K  S  C  N
tccatcgttgactgtatttcctggttagaagtattctgcgccgagctgacatcccgcattcaggatcttgaacaagagtat
S  I  V  D  C  I  S  W  L  E  V  F  C  A  E  L  T  S  R  I  Q  D  L  E  Q  E  Y
aacaagattgtcatccctcgtacagtctcgatctcactgaaaactaaatcgtacgaagtgtaccgtaagtcagggccggtg
N  K  I  V  I  P  R  T  V  S  I  S  L  K  T  K  S  Y  E  V  Y  R  K  S  G  P  V
gcctacaagggcatcaatttcaaagccacgagttattgaaagtcgggatcaaatttgtaaccgaccttgacattaaaggg
A  Y  K  G  I  N  F  Q  S  H  E  L  L  K  V  G  I  K  F  V  T  D  L  D  I  K  G
aaaaataaatcctactatccgttaacgaagctgtctatgaccattactaacttcgacatcatcgatttgcaaaaaactgtt
K  N  K  S  Y  Y  P  L  T  K  L  S  M  T  I  T  N  F  D  I  I  D  L  Q  K  T  V
gtggacatgtttgggaaccaagtacacacatttaagtcctcggcgggcaaagaggacgaggagaagacaactagcagtaag
V  D  M  F  G  N  Q  V  H  T  F  K  S  S  A  G  K  E  D  E  E  K  T  T  S  S  K
gcggatgaaaagacaccgaaactggaatgttgtaaatatcaagtgactttcacggaccaaaaagcacttcaagagcatgct
A  D  E  K  T  P  K  L  E  C  C  K  Y  Q  V  T  F  T  D  Q  K  A  L  Q  E  H  A
gactaccacctggctttaaagctttctgagggtctgaatggagcggaggagagcagtaaaaatttgtcctttggcgaaaag
D  Y  H  L  A  L  K  L  S  E  G  L  N  G  A  E  E  S  S  K  N  L  S  F  G  E  K
cgcttgctgttctctcgtaaacgtccaaacagtcaacacactgctactccccagaaaaagcaggttacttcatcaaaaaat
R  L  L  F  S  R  K  R  P  N  S  Q  H  T  A  T  P  Q  K  K  Q  V  T  S  S  K  N
attcttagtttcttcactcgtaaaaaatga
I  L  S  F  F  T  R  K  K  -
```

Supplementary Figure 1: Codon-optimised DNA and amino acid sequence for Sloppymerase. The enzyme features a HIS-tag at the 5’ end (yellow), a 5’-3’ exonuclease activity domain from *E. coli* DNA polymerase I (green), a 4xTGS spacer (pink), and DNA polymerase η from *S. cerevisiae* (turquoise).

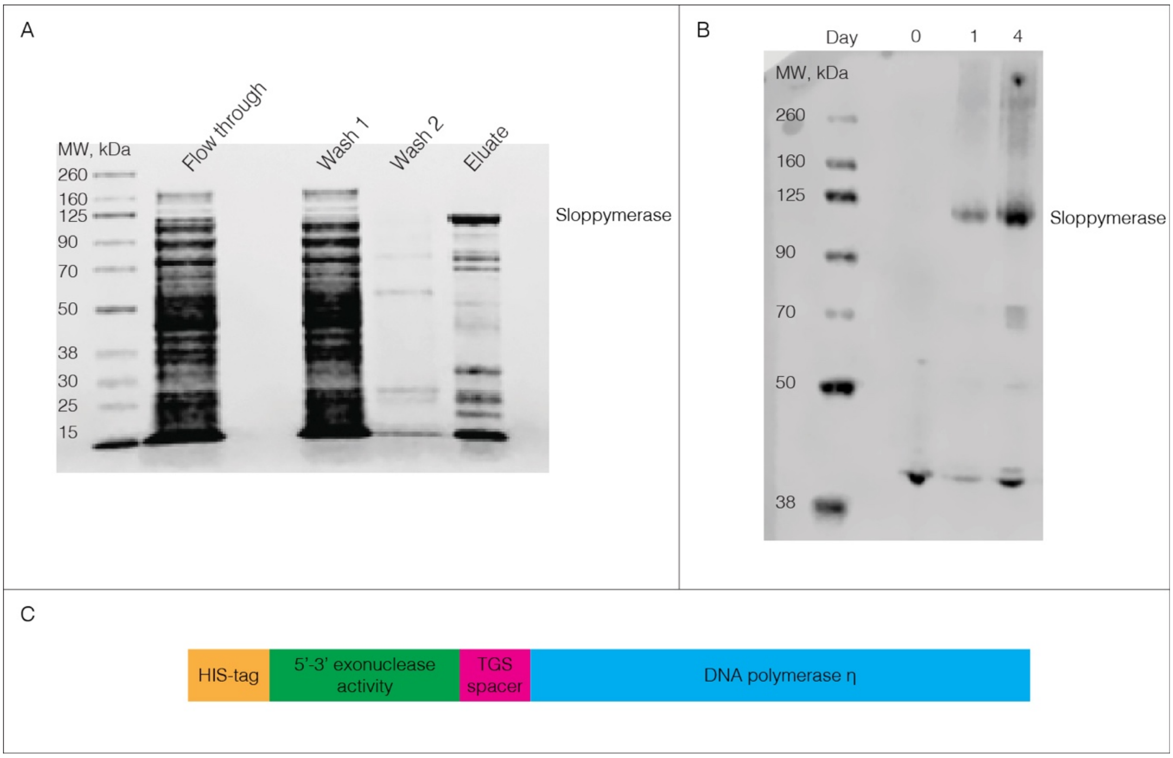

Supplementary figure 2: (A) Coomassie blue staining of flow-through, wash and final eluate from purification of histidine-tagged Sloppymerase with immobilized metal affinity chromatography. **(B)** Western blot staining for Sloppymerase (expected size 110kDa) with RAD30 antibody of *E. coli* lysate after induction with L-arabinose for either 0, 1 or 4 days. **(C)** A graphic representation of the different domains of Sloppymerase with HIS-tag (yellow), 5'-3' exonuclease activity (green), TGS spacer (pink) and DNA polymerase η (blue).

Methods:
For the western blot, *E. coli* Top 10 containing the Sloppymerase vector was cultured in LB medium containing 100 µg/ml ampicillin at 37°C for either 0 days, 1 day or 4 days. Every day, the culture was diluted 1:100,000 to maintain the bacteria in log phase. The cultures were induced with 0.02% L-arabinose on day 0, day 1, or day 4 and grown until the optical density at 600 nm (OD_{600}) reached 0.5. Before sampling, the samples from each culture were pelleted and lysed in 1.5× NuPAGE™ LDS Sample Buffer, supplemented with 20 mM dithiothreitol (DTT), and then denatured at 95 °C for 5 minutes. The samples were run on a NuPAGE Novex 4-12% Bis-Tris gel before being transferred to a PVDF-FL membrane. Chameleon Duo Pre-stained Protein Ladder (LI-COR Biosciences) was used for size reference. After the transfer, the membrane was washed with tris-buffered saline (TBS) and blocked for 2 hours at room temperature in Intercept blocking buffer (LI-COR Biosciences, diluted 1:3 in TBS). The membrane was incubated overnight at 4 °C with a rabbit polyclonal antibody against RAD30 (Catalog # PAB8981 (Abnova)) diluted 1:500 in blocking buffer. Thereafter, the membrane was washed three times for 10 minutes with 0.05% Tween-20 in TBS, followed by a 1-hour incubation with secondary antibody IRDye800 CW donkey anti-rabbit (LI-COR Biosciences) diluted in blocking buffer, and subsequently washed three times for 10 minutes with 0.05% Tween-20 in TBS. The membrane was scanned at 700 nm and 800 nm with an Odyssey® Fc imaging system (Li-Cor).

```
mhhhhhhenl yfqgmVQIPQ NPLILVDGSS YLYRAYHAFP PLTNSAGEPT GAMYGVLNML
RSLIMQYKPT HAAVVFDAKG KTFRDELFEH YKSHRPPMPD DLRAQIEPLH AMVKAMGLPL
LAVSGVEADD VIGTLAReae kAGRPVLIST GDKDMAQLVT PNITLINTMT NTILGPEEVV
NKygvppeli idflALMGDS SDNIPGVPGV GEKTAQALLQ GLGGLDTLYA EPEKiaglsf
rgaktmaakL EQNKEVAYLS YQLATIKtdv eleltceqle vqqpaAEELL GLFKkyefkR
WTADVEAGKW LQAKGAKPAA KPQETSVADE APEVTATTGS TGSTGSTGSM SKftwkelIQ
LGSPSKayes slaciahIDM NAFFAQVEQM Rcglskedpv vcvqwnsiia vsyaarkygi
srMDTIQEAL KKcSNLIPIH TAVFKKGEDF WQYHDGCGSW VQDPAKQISV EDHKvslepy
rresrkalki fksacdlver asiDEVFLDL GRicfnMLMF DNEYELTGDL KlkDALSNIR
EAFIGGNYDI NSHLPLIPEK ikSLKFEGDV FNPEGRdlit dwddvilalg sqvckgirds
ikdilgytts cglsstknvc klasnykkPD AQTIVKndcl ldfldcgkfe itSFWTLGGV
LGKELIDVLD LPHENSIKhi rETWPDNAGQ LKEFLDAKVK QSDYDRSTSN IDPLKTADLA
EKLFKlsrGR YGLPLSSRPV VKsmmsnknl rgkscnsivd ciswlevfca eltsrIQDLE
QEYNKIVIPR TVSISLKtks yevyrkSGPV AYKGINFQSH ELLKvgikFV TDLDIKGKnk
SYYPLTKLSM TITNFDIIDL QKTVVDMFGN QVHTFKssag kedeekttss kadektpkle
cckyQVTFTD QKALQEHADY HLALKLSEGL NGAEESSKNL SFGEKRllfs rkrpnsqhta
tpqkkQVTSS KNILSFFTRk k
```

Supplementary figure 3: Results from LC-MS analysis confirmed the successful expression of Sloppymerase in *E. coli*.

69% of peptides from the predicted amino acid sequence (in red) were detected in the purified sample of Sloppymerase.

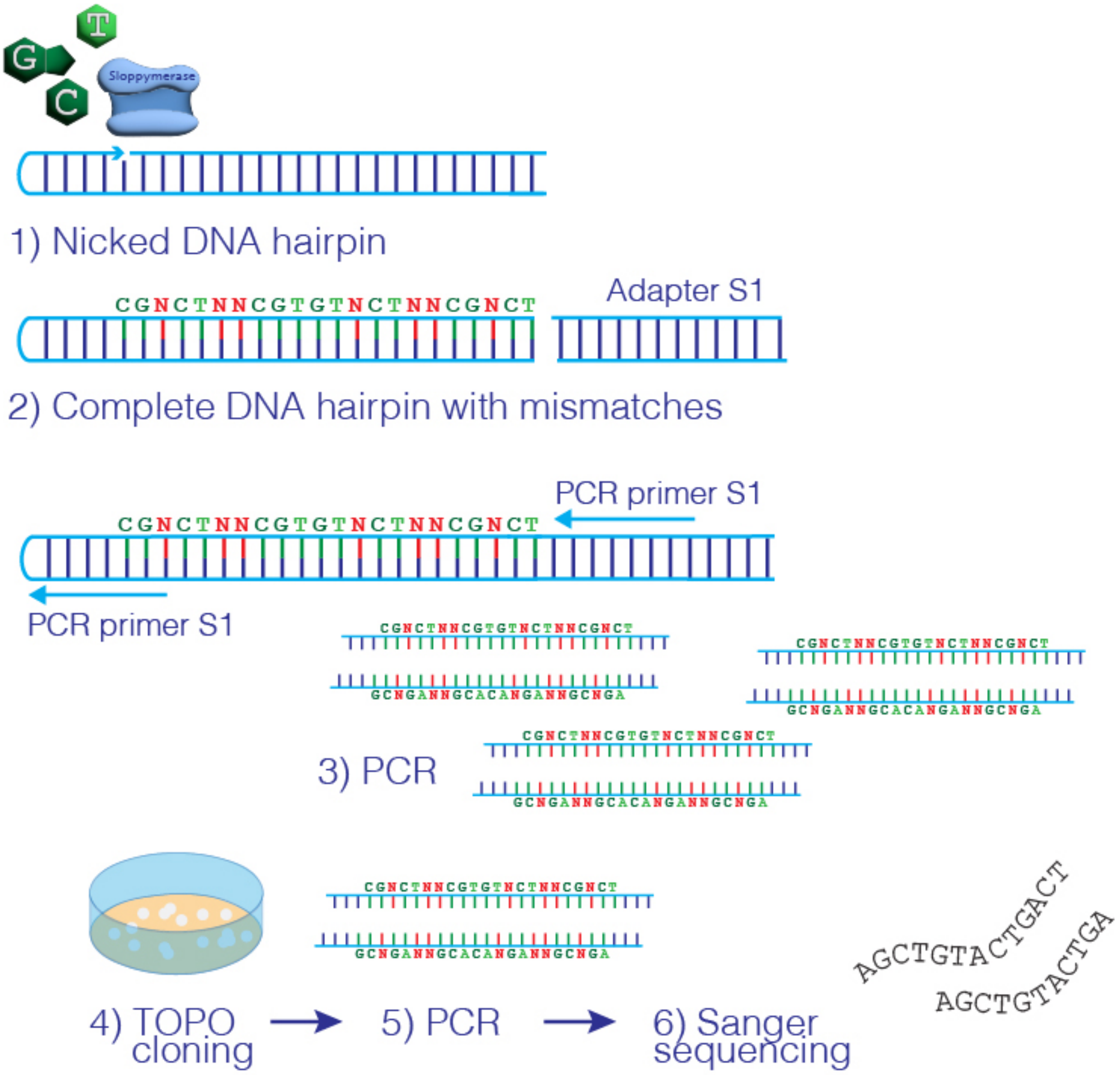

Supplementary figure 4: Schematic presentation of experimental design for TOPO cloning of Sloppymerase-treated hairpin

Supplementary Table 1: Genes differentially expressed by TGFβ treatment

Symbol	gene_id	log2FoldChange	DMSO	TGFß treatment
PLAUR	ENSG00000106366.9	5.00428238974659	31.2097889865832	1032.77728617607
CYP24A1	ENSG00000177606.9	3.97006682111833	7.22541738596139	127.904222204769
SNAI2	ENSG00000261052.6	3.88868088522766	0.29002022563095	18.1075986062729
FSTL3	ENSG00000264462.1	3.75065020253154	0	12.4604076952359
WNT11	ENSG00000138623.10	3.54920838532678	5.92082813571836	80.0170171412028
BAMBI	ENSG00000166106.4	3.09971904885024	0.878088918432808	15.0999512866516
IL11	ENSG00000183054.13	2.90900568769423	2.80010011960816	27.5425656404135
GADD45B	ENSG00000198959.12	2.80458840348343	1.20423623099356	14.4000939938714
SMAD7	ENSG00000203883.7	2.75271198345524	0.602118115496782	9.79800209892279
BMF	ENSG00000115844.11	2.61295481995903	4.4155328469764	32.1298120776364
SERPINE1	ENSG00000134107.5	2.52661684667545	57.5022800299427	336.101162908503
NEDD9	ENSG00000171223.6	2.45688238681786	155.045414740421	855.734598899426
DLX2	ENSG00000169093.16	2.44159755234294	0	4.43242952094126
CCN2	ENSG00000137331.12	2.441262020024	13.3469515601787	76.9206788155692
HS3ST2	ENSG00000142178.9	2.43196279848132	45.7564358036344	251.31039796545
PMEPA1	ENSG00000280614.1	2.40162362542136	0	4.28397494368486
CDKN1A	ENSG00000095752.7	2.32339014338855	0.903177173245174	8.52553429386788
SOX9	ENSG00000125740.15	2.31633536378306	8.75580092951571	47.5902747012568
FOSB	ENSG00000136603.15	2.19783883569121	241.725360205401	1112.60345435431
TMCC2	ENSG00000070404.10	2.16995580029244	25.5900199086133	118.657644029167
BHLHE40	ENSG00000101665.10	2.16690711038077	28.4751692120353	131.361093075168
SKIL	ENSG00000189431.8	2.16134327064955	10.3363609826948	49.7110755841451
PIM1	ENSG00000278709.2	2.04155484813615	3.08585534192101	15.8210163761827
IER3	ENSG00000280441.4	2.02917430496678	1.49997657872173	9.20418378989717
SEMA7A	ENSG00000175505.11	2.01790885788057	3.93885600554145	19.00218588882
SIK1	ENSG00000168140.5	1.98903717127301	0.827912408808076	6.25630004151996
CCN1	ENSG00000255774.3	1.95304753351093	2.83497279379735	13.8486912783476
WNT9A	ENSG00000259132.1	1.90213999655964	0	2.73767206698213
ACKR3	ENSG00000269693.1	1.88145385884567	0	2.68446170493409
ATF3	ENSG00000162772.18	1.87774136969768	18.7660145996497	71.6399586323881
HEY1	ENSG00000259976.3	1.83785243202932	0	2.57477498013835
ADAMTS15	ENSG00000122254.7	1.81523752969082	4.34026808253931	17.7933414740178
VASN	ENSG00000118523.6	1.7992875870189	1.50529528874196	7.71963801733311
ASMTL	ENSG00000137193.14	1.79033864642246	39.8903251516618	140.43803008456
MN1	ENSG00000275993.4	1.77575171024188	2.01160135911031	9.31221630654633
JUNB	ENSG00000125398.8	1.76832283164884	39.8903251516618	138.296042612718
MYEOV	ENSG00000287856.1	1.76323376066901	0.392430481775028	3.72671887625781
CLCF1	ENSG00000111859.17	1.75037933063309	67.3619641712025	229.001789316382
JUN	ENSG00000133069.17	1.74605472537852	4.74168015953716	18.2598917947412
EGR3	ENSG00000164683.19	1.73468856212422	1.50532037699677	7.33789767581664
PHLDA2	ENSG00000011422.12	1.72279149633732	4.8671465218538	18.3659519862925
RGPD6	ENSG00000143816.8	1.71261578530032	29.1023755823445	97.6619040379642
NOG	ENSG00000095739.11	1.69939665407854	3.96394426035382	15.1211590834025
RASSF10	ENSG00000179388.9	1.68218179095442	1.12897146655647	5.83214410650166
TGM2	ENSG00000124225.16	1.67066908363601	280.637218331125	895.626464587897
SOX18	ENSG00000019186.10	1.6706028226126	3.21129661598284	12.4065610992854
MAFB	ENSG00000279967.1	1.65167742813471	0	2.14198747184243
LINC02551	ENSG00000204103.4	1.60233335946876	0.67738287993388	4.09310477292662
LINC02747	ENSG00000085741.14	1.59579467810021	2.03214863980164	8.16500174910233
	ENSG00000181649.9	1.59429520078359	9.85968414125981	31.7904873296218
	ENSG00000144476.6	1.58962375236808	34.5716151314403	106.060191551327
SULT1A3	ENSG00000124762.15	1.58867283321954	99.0986065088454	300.069116228698
MIR3648-2	ENSG00000169184.7	1.56426564395244	4.46570935660114	15.1635746769043
	ENSG00000019549.13	1.56285632944013	38.0588825503591	114.394855674436
	ENSG00000183691.6	1.56187643463278	0.200706038498927	2.54493561010982
NKILA	ENSG00000099860.10	1.53910541156729	15.7052726007959	47.5479015233485
	ENSG00000104081.14	1.51922206180713	48.7213908456146	141.519627718857
	ENSG00000142871.18	1.51394915958786	17.1352780368459	50.7926732184417
	ENSG00000172927.9	1.50653705258556	9.18230126132593	27.930689528752
	ENSG00000254842.7	1.50193531224683	0.401412076997855	2.96911275292487

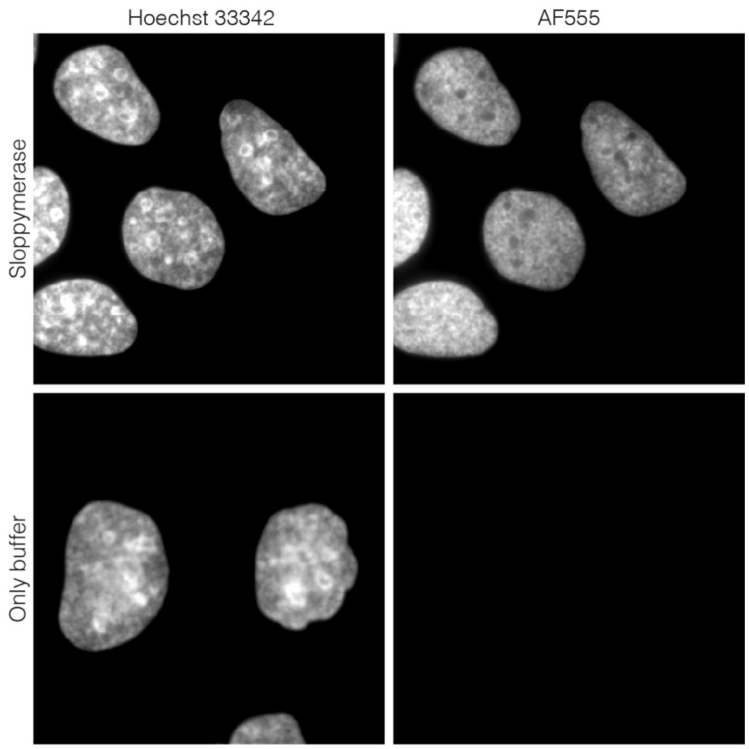

Supplementary figure 5: SSB labelling in fixed cells
Sloppymerase labeling of SSBs in fixed HaCaT cells treated with Nt.BsmAI is shown. The nuclei were stained with Hoechst 33342 as indicated in the left panel. The incorporation of AF555-dUTP in the presence of Sloppymerase is shown in the upper right panel. The absence of AF555-dUTP incorporation when Sloppymerase was omitted (buffer only) is shown in the lower right panel.

Methods:
The SSB labelling was conducted as described in Bivehed *et al.* [1]. Briefly, HaCaT cells were seeded in chamber slides, cultivated for 24 hours and thereafter fixed with ice-cold ethanol (70%) for 30 minutes, followed by ethanol washes (96%-99.5%). To induce SSBs, the slides were subjected to 125 mU/µl Nt.BsmAI in 1x CutSmart buffer for 1 hour at 37°C followed by 2x5 minutes washes with TBS. For SSB detection, the slides were incubated with 0,315 µg/µl Sloppymerase diluted in 1x NEBuffer 2.1, 0.1 mM $MnCl_2$, 0.1 mM dATP/dGTP/dCTP, 0.08 mM dTTP, 0.02 mM Aminoallyl-dUTP-XX-AF555 for 60 minutes at 37 °C in a moisture chamber. Thereafter, the slides were subjected to a series of washes: 3 times quickly rinsed with H_2O, 2 times for 5 minutes with TBS-tween, 5 minutes with 10 µg/ml Hoechst 33342 diluted in TBS-tween, and finally 2 times for 5 minutes with TBS. The slides were mounted with ProLong Glass antifade (Thermofisher Scientific) and cured overnight before being imaged. All experiments were conducted independently three times, and at least three images were acquired per replicate.
Images were acquired using a Zeiss Imager M2 microscope equipped with a Plan-Apochromat 63X/1.4 Oil objective, an HXP 120 V light source, a Hamamatsu C11440 camera, and Zen 2 software (Blue Edition). Cube filter sets 43HE and 49 were used (all from Zeiss). Signal strength was equally enhanced for visualization purposes.

Supplementary Reference
1. Bivehed, E. et al. Visualizing DNA single- and double-strand breaks in the Flash comet assay by DNA polymerase-assisted end-labelling. *Nucleic Acids Res* **52**, e22 (2024).
