# Supplementary material for: Precise mapping of single-stranded DNA breaks by sequence-templated erroneous DNA polymerase end-labelling
Source: Nat Commun. 2025 Aug 4;16:7130. doi: 10.1038/s41467-025-62512-4 (PMC12322144; doi:10.1038/s41467-025-62512-4)

Source data Figure 2A

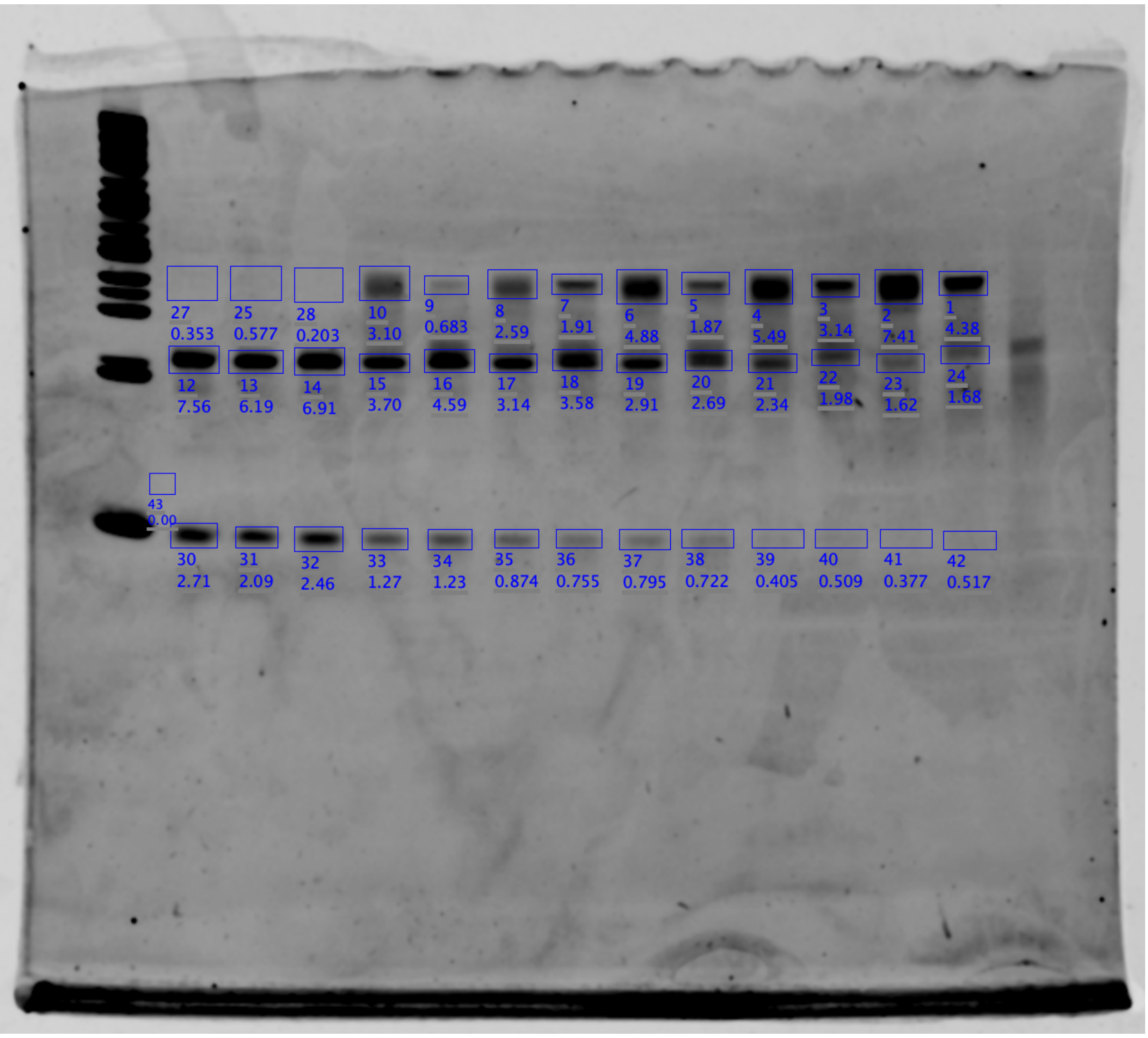

Source data Figure 2B

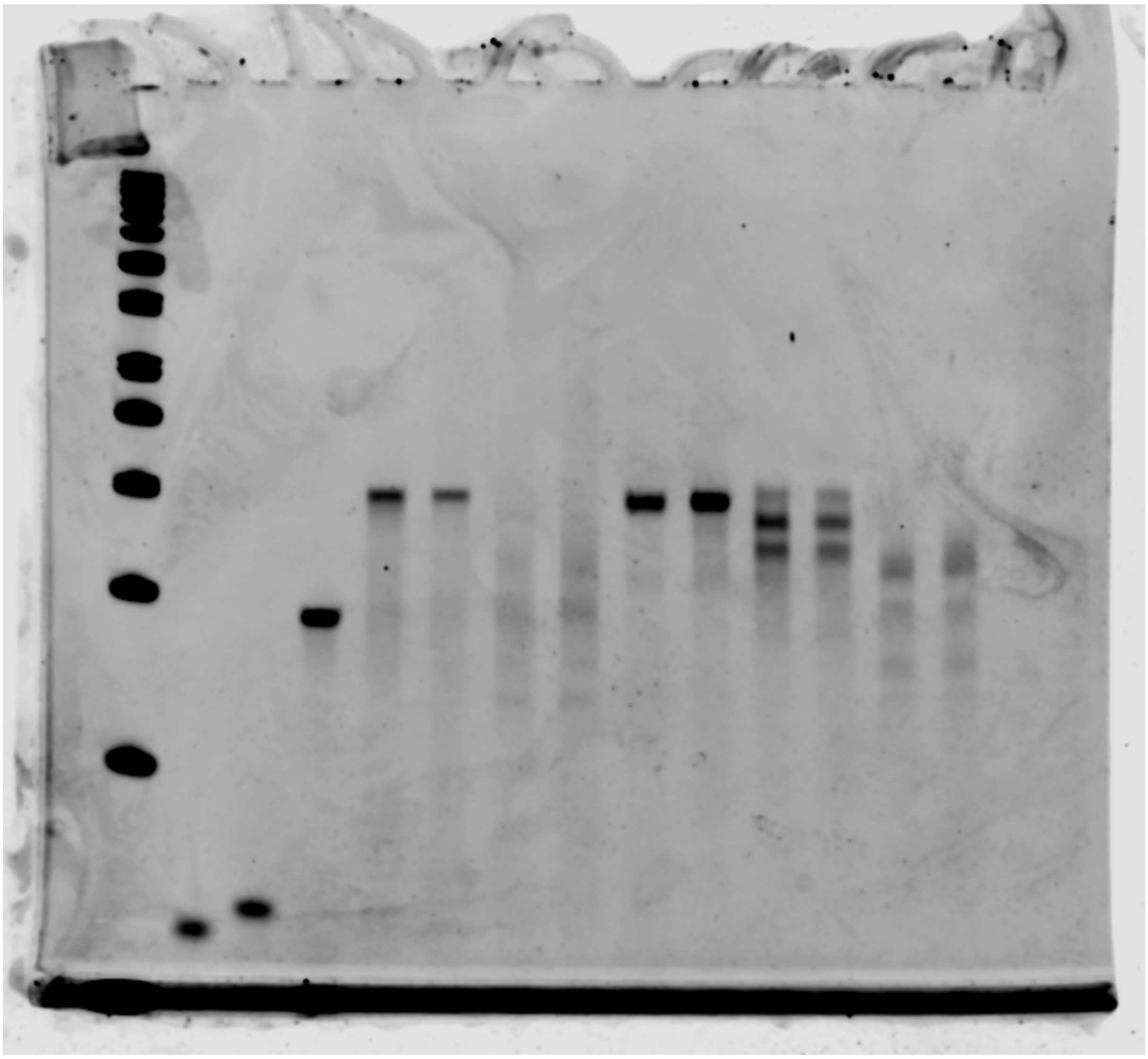

Source data Figure 5B

Well	Fluor	Target	Sample	Cq	Cq Mean	Cq Std. Dev
A01	SYBR	GAPDH	DMSO	19,28	19,25	0,037
A02	SYBR	GAPDH	DMSO	19,21	19,25	0,037
A03	SYBR	GAPDH	DMSO	19,26	19,25	0,037
A04	SYBR	GAPDH	BMH-21	18,88	18,87	0,033
A05	SYBR	GAPDH	BMH-21	18,90	18,87	0,033
A06	SYBR	GAPDH	BMH-21	18,83	18,87	0,033
A07	SYBR	GAPDH	TGFb	19,21	19,27	0,058
A08	SYBR	GAPDH	TGFb	19,32	19,27	0,058
A09	SYBR	GAPDH	TGFb	19,27	19,27	0,058
A10	SYBR	GAPDH	BMH+TGFb	18,79	18,86	0,084
A11	SYBR	GAPDH	BMH+TGFb	18,84	18,86	0,084
A12	SYBR	GAPDH	BMH+TGFb	18,95	18,86	0,084
B01	SYBR	SERPINE1	DMSO	26,43	26,34	0,082
B02	SYBR	SERPINE1	DMSO	26,29	26,34	0,082
B03	SYBR	SERPINE1	DMSO	26,29	26,34	0,082
B04	SYBR	SERPINE1	BMH-21	27,20	27,19	0,045
B05	SYBR	SERPINE1	BMH-21	27,23	27,19	0,045
B06	SYBR	SERPINE1	BMH-21	27,14	27,19	0,045
B07	SYBR	SERPINE1	TGFb	21,11	21,05	0,047
B08	SYBR	SERPINE1	TGFb	21,02	21,05	0,047
B09	SYBR	SERPINE1	TGFb	21,04	21,05	0,047
B10	SYBR	SERPINE1	BMH+TGFb	24,72	24,75	0,162
B11	SYBR	SERPINE1	BMH+TGFb	24,60	24,75	0,162
B12	SYBR	SERPINE1	BMH+TGFb	24,92	24,75	0,162
C01	SYBR	SMAD7	DMSO	26,26	26,25	0,049
C02	SYBR	SMAD7	DMSO	26,19	26,25	0,049
C03	SYBR	SMAD7	DMSO	26,29	26,25	0,049
C04	SYBR	SMAD7	BMH-21	25,60	25,67	0,071
C05	SYBR	SMAD7	BMH-21	25,75	25,67	0,071
C06	SYBR	SMAD7	BMH-21	25,66	25,67	0,071
C07	SYBR	SMAD7	TGFb	23,95	23,98	0,024
C08	SYBR	SMAD7	TGFb	24,00	23,98	0,024
C09	SYBR	SMAD7	TGFb	23,99	23,98	0,024
C10	SYBR	SMAD7	BMH+TGFb	23,99	24,00	0,016
C11	SYBR	SMAD7	BMH+TGFb	24,00	24,00	0,016
C12	SYBR	SMAD7	BMH+TGFb	24,02	24,00	0,016
D01	SYBR	IL11	DMSO	30,60	30,44	0,161
D02	SYBR	IL11	DMSO	30,28	30,44	0,161
D03	SYBR	IL11	DMSO	30,44	30,44	0,161
D04	SYBR	IL11	BMH-21	30,20	30,17	0,138
D05	SYBR	IL11	BMH-21	30,02	30,17	0,138
D06	SYBR	IL11	BMH-21	30,30	30,17	0,138
D07	SYBR	IL11	TGFb	28,34	28,41	0,189
D08	SYBR	IL11	TGFb	28,62	28,41	0,189
D09	SYBR	IL11	TGFb	28,26	28,41	0,189
D10	SYBR	IL11	BMH+TGFb	30,07	30,40	0,355
D11	SYBR	IL11	BMH+TGFb	30,35	30,40	0,355
D12	SYBR	IL11	BMH+TGFb	30,78	30,40	0,355
F01	SYBR	JUNB	DMSO	23,88	23,77	0,102
F02	SYBR	JUNB	DMSO	23,75	23,77	0,102
F03	SYBR	JUNB	DMSO	23,68	23,77	0,102
F04	SYBR	JUNB	BMH-21	24,44	24,38	0,054
F05	SYBR	JUNB	BMH-21	24,36	24,38	0,054
F06	SYBR	JUNB	BMH-21	24,33	24,38	0,054
F07	SYBR	JUNB	TGFb	21,10	21,11	0,025
F08	SYBR	JUNB	TGFb	21,13	21,11	0,025
F09	SYBR	JUNB	TGFb	21,09	21,11	0,025
F10	SYBR	JUNB	BMH+TGFb	23,38	23,42	0,032
F11	SYBR	JUNB	BMH+TGFb	23,43	23,42	0,032
F12	SYBR	JUNB	BMH+TGFb	23,45	23,42	0,032
H01	SYBR	CHD4	DMSO	23,47	23,44	0,055
H02	SYBR	CHD4	DMSO	23,38	23,44	0,055
H03	SYBR	CHD4	DMSO	23,47	23,44	0,055
H04	SYBR	CHD4	BMH-21	23,11	23,09	0,024
H05	SYBR	CHD4	BMH-21	23,06	23,09	0,024
H06	SYBR	CHD4	BMH-21	23,10	23,09	0,024
H07	SYBR	CHD4	TGFb	23,33	23,40	0,090
H08	SYBR	CHD4	TGFb	23,37	23,40	0,090
H09	SYBR	CHD4	TGFb	23,50	23,40	0,090
H10	SYBR	CHD4	BMH+TGFb	23,09	23,15	0,062
H11	SYBR	CHD4	BMH+TGFb	23,15	23,15	0,062
H12	SYBR	CHD4	BMH+TGFb	23,22	23,15	0,062

Source data Supplementary Figure 2A

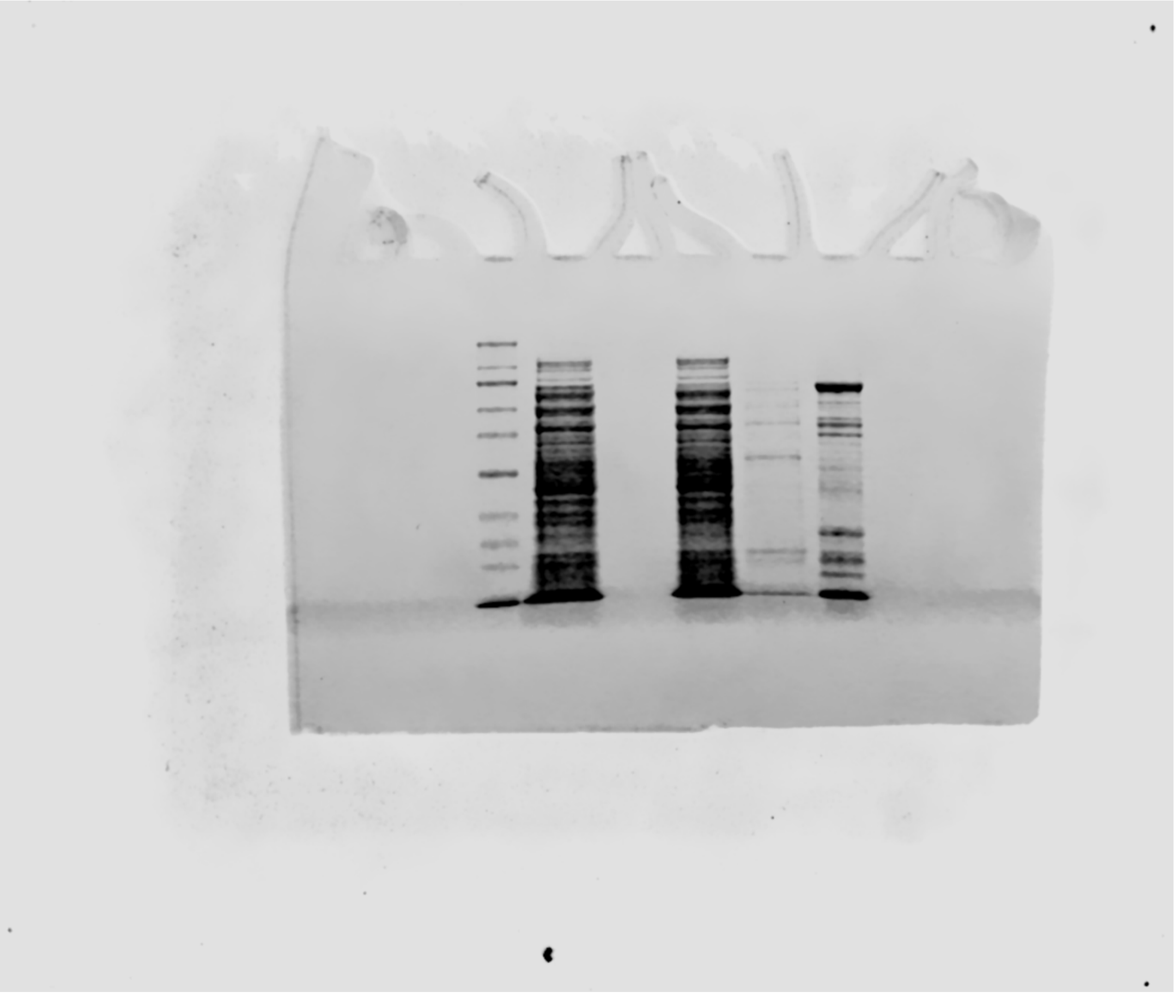

Source data Supplementary Figure 2B

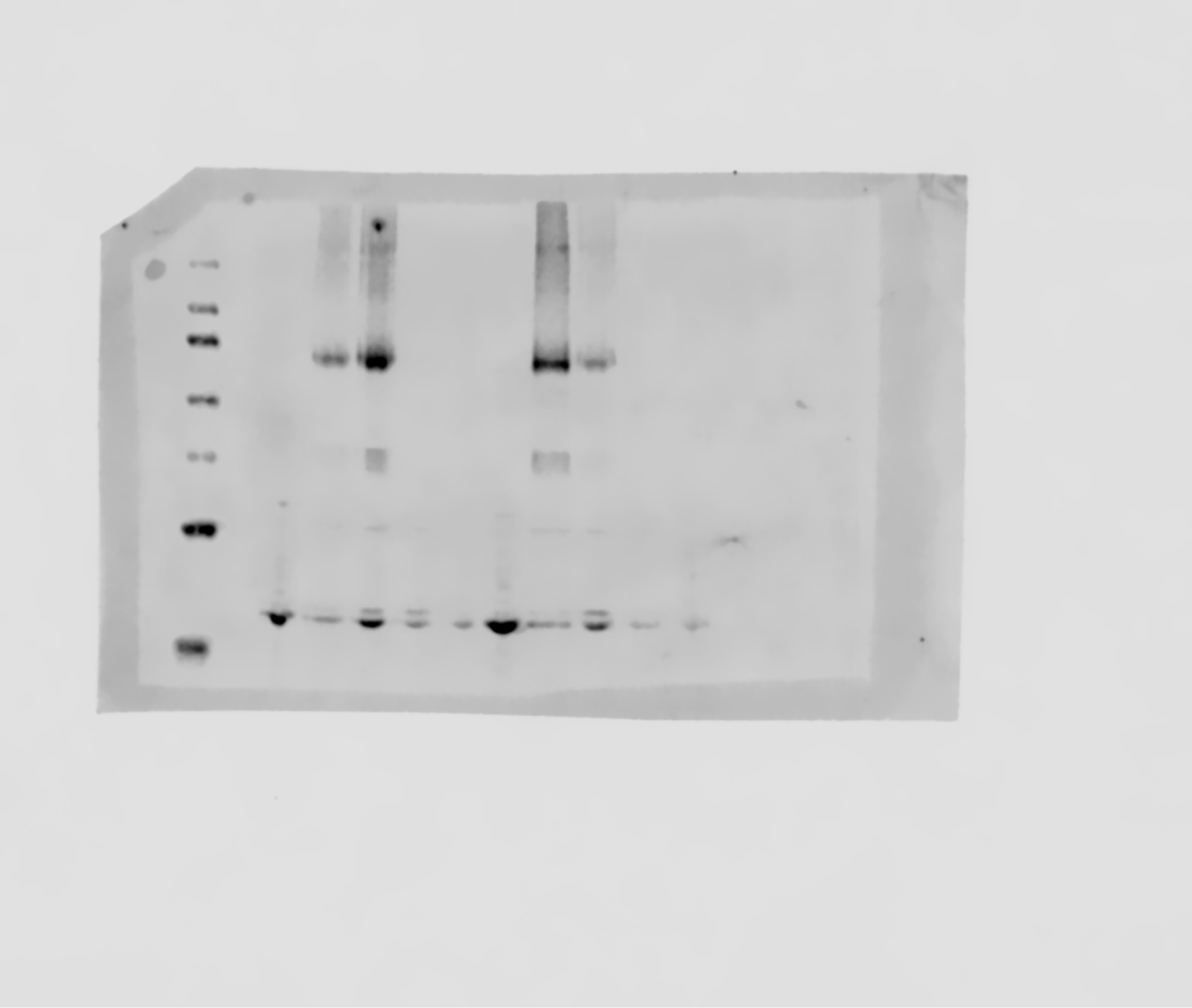

Supplement: Supplementary file 4 — Source Data [file 41467_2025_62512_MOESM4_ESM.pdf]
